# From mechanisms to function: an integrated framework of animal innovation

**DOI:** 10.1098/rstb.2015.0195

**Published:** 2016-03-19

**Authors:** Sabine Tebbich, Andrea S. Griffin, Markus F. Peschl, Kim Sterelny

**Affiliations:** 1 Department of Behavioural Biology, University of Vienna, Vienna 1090, Austria; 2 School of Psychology, University of Newcastle, Callaghan, New South Wales 2308, Australia; 3 Department of Philosophy and Cognitive Science Research Platform, University of Vienna, Vienna 1010, Austria; 4 School of Philosophy, Research School of the Social Sciences, Australian National University, Canberra, Australian Capital Territory 0200, Australia

**Keywords:** innovation, behavioural innovation, play, evolution, taxonomic radiation, embodied cognition

## Abstract

Animal innovations range from the discovery of novel food types to the invention of completely novel behaviours. Innovations can give access to new opportunities, and thus enable innovating agents to invade and create novel niches. This in turn can pave the way for morphological adaptation and adaptive radiation. The mechanisms that make innovations possible are probably as diverse as the innovations themselves. So too are their evolutionary consequences. Perhaps because of this diversity, we lack a unifying framework that links mechanism to function. We propose a framework for animal innovation that describes the interactions between mechanism, fitness benefit and evolutionary significance, and which suggests an expanded range of experimental approaches. In doing so, we split innovation into factors (components and phases) that can be manipulated systematically, and which can be investigated both experimentally and with correlational studies. We apply this framework to a selection of cases, showing how it helps us ask more precise questions and design more revealing experiments.

## 1. Background

A changing world both poses challenges to, and provides new opportunities for, an organism. Behavioural innovations allow humans and other animal species to respond adaptively to a changing world. As such, they provide a route for animals to not only deal with challenges, but also to capitalize on them, and even create novel opportunities. By innovating, animals may encounter new selective environments, which in turn may offer yet more novel opportunities. In this way, behavioural innovations can initiate an evolutionary runaway process that paves the way for phylogenetic radiation [1].

Work on animal innovations has exploded since Reader & Laland's [2] keystone collection re-energized interest in the topic. Since then, research on innovation ramified. On the functional level, large-scale multi-species analyses have related anecdotal reports of novel feeding behaviours to ecological and evolutionary parameters (see reviews [3–5]). These studies aim to determine the environmental drivers, the adaptive significance and the evolutionary consequences of innovations, while making few assumptions about mechanisms (reviewed by [3]). Innovation counts correlate positively with occupation of harsh and changing environments and colonization of novel environments, as well as with lineage diversification [6–10]. Diet generalists have a higher frequency of technical foraging innovations (e.g. novel searching and handling techniques) [11] and food-type innovations, whereas habitat generalists have only higher food-type innovations ([12], but see [13]). When studies rely on anecdotal reports, there is of course a possibility that these are shaped by reporting biases, but substantial effort has been invested in accounting for research and observation biases, increasing confidence that taxonomic differences in innovation counts are not purely attributable to these confounding variables [3,5].

Spurred along by these macro-ecological level findings, an experimental programme aims to identify the proximate mechanisms of innovation [14,15]. Specific species are challenged with tasks designed to elicit feeding innovations, with the aim of identifying the mechanisms that promote innovation. This experimental approach presumes that the mechanisms deployed in the experimental task also explain innovation in the wild. These studies have led to a dichotomy between *simple* mechanisms whereby animals generate random behavioural variants and learn quickly about their consequences, and *complex* mechanisms whereby animals employ higher-order cognitive mechanisms such as causal reasoning and abstract rules to solve novel problems [16–18]. Studies of innovation guided by simple mechanisms have shown that both within and across avian species, innovation propensity increases with more diverse motor actions, especially if the agent samples regularly across its full range of motor possibilities [19–21]. Research on complex mechanisms has focused on large-brained species and challenging innovations. For example, research has shown that New Caledonian crows (*Corvus moneduloides*) can identify the features of objects that make them useful to solving particular kinds of problems, store these in memory and use this information in subsequent problem-solving opportunities [22]. It has also become clear that cognitive processes involved in innovation are moderated by general-purpose cognitive mechanisms such as attention and inhibitory control, and by motivational systems such as perseverance, reactions to novelty (neophilia and neophobia) and play [23–25]. We suggest that proximate-level research on innovation would be enhanced by incorporating insights from the embodied approach to the study of cognition [26–28]. This approach recognizes the importance not just of cognitive mechanisms in the strict sense (associative learning and other cognitive capacities) but also of an agent's perceptual capacities, motor capacities and motivational mechanisms. Innovation is influenced by the interaction between the agent's capacities, both cognitive and physical, and the options made available (‘the affordances’) [29] by the target of innovative action and by the broader environment.

Research on innovation is rich and diverse, but it lacks a *unifying* and *organizing* framework that relates the diversity of behavioural phenomena to their underpinning mechanisms on the one hand, and to their potential evolutionary consequences on the other. Kolodny *et al.* [30] develop a more synthetic and theoretically oriented approach, but the emphasis of that paper is on the computational organization of forms of learning that are likely to lead to innovation. The approach developed here complements that approach, linking a range of cognitive mechanisms to motor capacities, and to environments that make innovation more probable.

Linking mechanism and function enables us to ask more precise questions and design more revealing experiments. Current experimental paradigms most probably shed light on only a subset of mechanisms, and therefore only on a subset of ways in which innovations can arise. This paper develops a more comprehensive framework and shows how it can generate new research directions.

## 2. Innovation: an integrated framework

**Figure 1.**
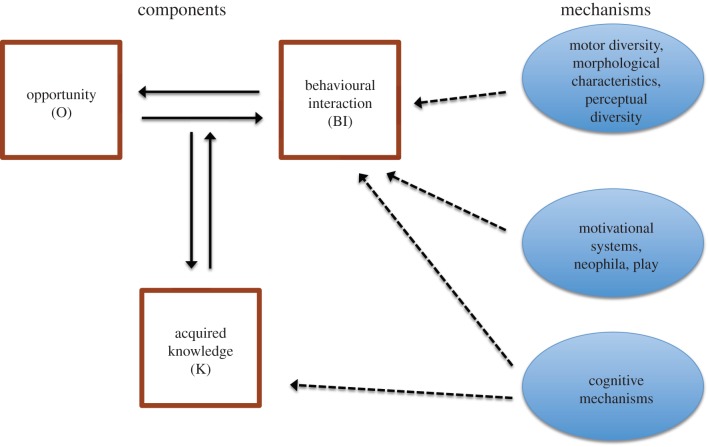
Components for a conceptual model of innovation. The three structural components are involved in the process of innovation: (1) the environmental opportunity (O), (2) the behavioural interaction (BI) and (3) the knowledge (K) that is acquired from a behaviour–opportunity interaction. The availability of opportunities depends upon an agent's perceptual mechanisms and motor repertoire, whereas the behavioural interactions and the acquired knowledge are shaped by the agent's cognitive mechanisms. The mechanisms enable BI and K (dotted lines). (Online version in colour.)

The premise of our framework is that the environment offers a multitude of as yet unexploited resources, which we refer to as ‘opportunities’. We regard an innovation as the discovery of a new behavioural interaction with the social or physical environment, tapping into an existing opportunity and/or creating a new opportunity. The first part of our framework decomposes innovation into three structural *components*. The components include (i) the environmental opportunity (O), (ii) the behavioural interaction (BI) and (iii) the knowledge (K) that is acquired from a behaviour–opportunity interaction (figure 1). The opportunities available to an agent depend upon its perceptual mechanisms and its motor repertoire. The behavioural interactions and the acquired knowledge are shaped by the agent's cognitive mechanisms (e.g. perception, long-term memory, response-outcome learning, causal reasoning, etc.) and its motivational systems (e.g. attention, neophilia, perseverance and play). We refer to all these determining processes as *mechanisms* (figure 1).

The three components interact with each another in various combinations: we propose *six phases* of the innovation process over which such an interaction can take place (table 1 and see below). The knowledge that is acquired during an interaction with an opportunity varies along a continuum from being tightly bound to the original opportunity to being gradually more decoupled from it. To the extent that the information is decoupled (i.e. more general), the agent is able to apply it to reach or create adjacent, as yet unexploited opportunities (for a similar idea of ‘adjacent possibilities’ see [31]). For example, several bird species have been observed to break nuts by dropping them on hard surfaces [32]. This new technique can be generalized to perceptually similar opportunities (dropping mussels) or to perceptually quite different opportunities (killing live prey by dropping them). As a consequence of gradual decoupling of acquired knowledge, and increased breath of access to new opportunities, phases have different evolutionary/fitness-enhancing potentials, as outlined below.

**Table 1.** Phases of innovation, the main mechanisms involved in each phase, the knowledge acquired during the process and the structural components involved. O, environmental opportunity; BI, behavioural interaction; K, knowledge that is acquired from this interaction.

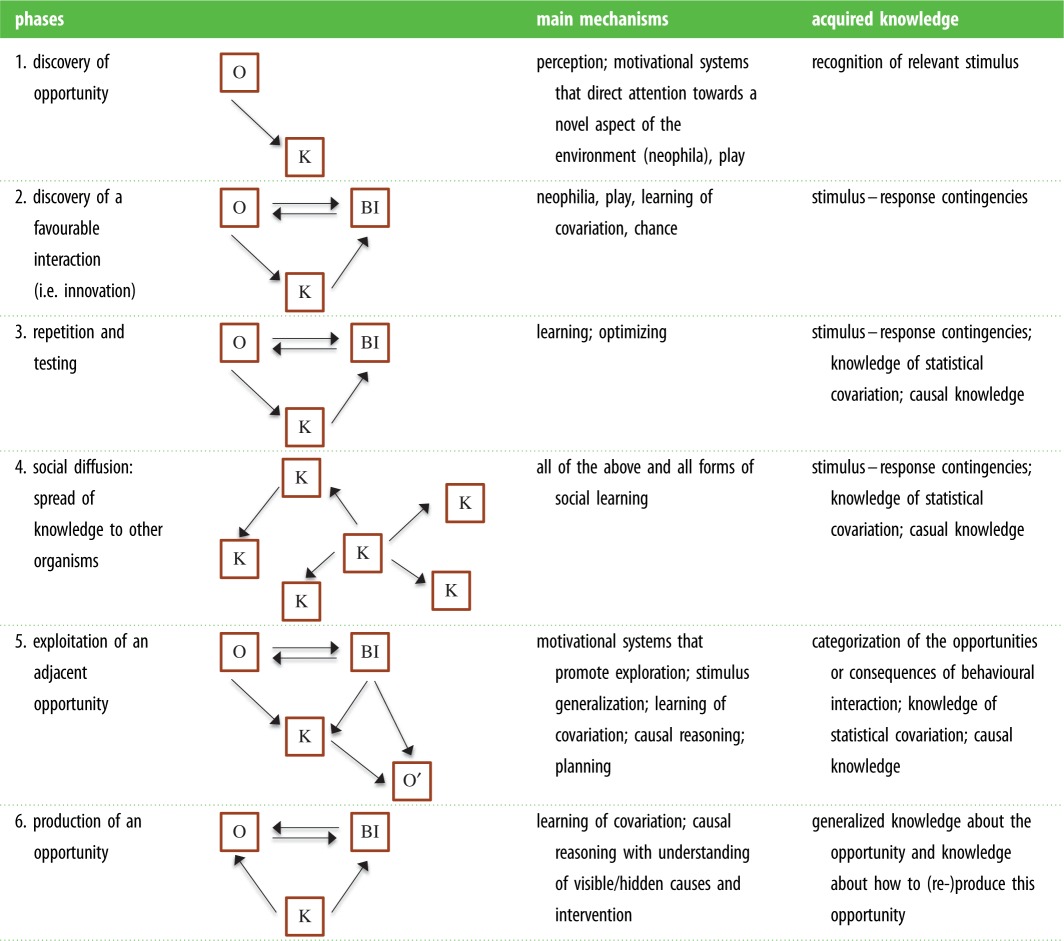

## 3. The components

### (a) Component 1: opportunity

We refer to a yet unexploited resource as an opportunity; it can be situated in various domains (e.g. food, predator avoidance, communication or social life). Opportunities may vary along various dimensions: (i) few to many affordances (e.g. hard-shelled prey may offer fewer opportunities to handle and access than prey without a shell), (ii) low-to-high degree of novelty, where novelty refers to the extent to which the new opportunity resembles those already exploited. For example, catching insects in artificial light by American redstarts (*Setophaga ruticilla*) [33] might be similar to catching insects in moonlight. (iii) Opportunities also vary in their potential fitness impact. For example, eating a new species of insect with a similar nutritional value to one already exploited might have a modest fitness impact. By contrast, discovering a fluid source in a very dry environment might yield very large survival and reproductive benefits. (iv) Opportunities also vary in the extent to which their potential is already recognized. The agent may know about the profitability of the resource, and so the innovations will involve techniques for harvesting that resource from previously inaccessible places (e.g. accessing honey with a stick by apes [34]). Overcoming neophobia is unlikely to be important for these innovations [34]. In cases where an opportunity's whose potential is unknown, innovation probably depends both on a lucky accident and overcoming neophobia (e.g. taking milk from a lactating elephant seal (*Mirounga leonine*) by the southern skua (*Stercorarius antarcticus*) [13]). (v) Finally, opportunities can vary in their perceptual and/or physical accessibility. Rewards can be fast and obvious (e.g. scavenging on road kill [13]). Alternatively, opportunities can be delayed and cryptic (e.g. feruginous hawks (*Buteo regalis*), using gunshots as a signal for prey availability, raiding gastric cavities of sea anemones by turnstones (*Arenaria interpres*) [13]). As potential benefits are unlikely to be known in cases where opportunities are cryptic and delayed, innovations are likely to depend, here too, on lucky accident and overcoming neophobia.

### (b) Component 2: behavioural interactions

Agents must interact with targets to exploit opportunities. As with opportunities, interactions vary in novelty. Some adapt or optimize an existing pattern of action to a new end: an animal that already uses a stick to reach inaccessible food uses it to reach an inaccessible part of its body. Some involve novel behaviours: for example, crimson rosellas (*Platycercus elegans*) have been observed catching insects on the wing [13] although they normally forage while climbing in bushes and trees. The possible range of interactions is strongly influenced by the breadth of the agent's behavioural repertoire, by its propensity to switch from one foraging technique to another and to develop new ones. In addition, the agent's motivational systems—its willingness to explore novel objects—are important in interactions with novel opportunities. Finally, an agent's capacity expands significantly if that agent can incorporate objects into its behaviour (e.g. all forms of tool use). This ability will be structured by the specific attributes of the agent's body. For instance, the active incorporation of objects into the behavioural interaction with the environment is easier for agents that can grab and manipulate them. So morphological features like hands and feet facilitate object exploration.

### (c) Component 3: acquired knowledge

We postulate that in establishing an innovation, agents acquire knowledge as a consequence of the discovery of, and interaction with an opportunity (phases 1 and 2, table 1). Success as well as failure may improve this knowledge [35]. Without such knowledge, the discovery of an opportunity and/or an interaction would either occur only once (i.e. never progress to phase 3, table 1) or would need to occur repeatedly by chance. Innovations of this kind are unlikely to progress beyond phase 3 (table 1).

Empirical findings from research in comparative cognition suggest that the nature of the acquired knowledge varies in form and content across species [36,37]. We expect to find a continuum, from knowledge that is connected to a particular and specific innovation opportunity (procedural/implicit knowledge, ‘knowing how’) to more general knowledge that can be applied more broadly to a variety of new opportunities (declarative/explicit knowledge, ‘knowing that’) [38–40], though this will come in degrees, depending on the range of circumstances in which the information can be used. The more the agent's encoding of the knowledge is declarative and generalized, the greater the flexibility, because it is available to influence behaviour in a variety of situations. In its simplest form, acquired knowledge will form straightforward conditioned stimulus–response associations (e.g. drop mussels). In some species, associative knowledge will become goal-directed (e.g. drop mussels to access the food contained within) [41]. Goal directionality results in an increase in response flexibility (e.g. do not drop mussels if they made you sick) [42].

Nevertheless, associative knowledge remains bound to the specific observable attributes of an agent–opportunity interaction. Under these circumstances, the discovery of adjacent opportunities (phase 5, table 1) relies upon perceptual attributes common to both the original and a new opportunity (e.g. drop any hard-shelled food without having had to discover each hard shell–food association individually). Association-based knowledge can expand, but only by responding to increasingly different variations of the original perceptual attributes (‘stimulus generalization’; e.g. drop all foods that cannot be immediately killed and eaten). Associative learning can therefore drive agents from discovering and repeating an initial opportunity to the discovery of adjacent opportunities through a chain of similarities (i.e. from phases 1–3 to phase 5, table 1).

However, innovating animals need not be bound to perceptible attributes of opportunities (e.g. in associative learning) nor restricted to knowledge of statistical covariation [43,44] (but see [45] for a sceptical view on sensitivity to statistical covariation in animals/humans). Knowledge can involve explicit knowledge of causal relationships; this implies greater capacities to make inferences about perceivable and unperceivable (hidden) causes and interventions [46–50]; the extent to which these more sophisticated forms of cognition have been important in animal innovation remains to be discovered. However, our framework does not suggest a sharp break between association-based knowledge of regularities and causal knowledge. We envisage that an accumulation of knowledge of covariation might form the basis for knowledge that goes beyond statistical regularities and becomes causal. Just as with novel opportunities and behavioural interactions, the content of causal knowledge will vary along multiple dimensions: (i) cross-domain applicability (e.g. causal knowledge is applied both in tool use and nest building); (ii) the temporal depth across which causal connections can be recognized (cause and effect that vary in their temporal proximity); (iii) complexity (i.e. can the agent understand causal interactions in which more than one cause is relevant?); (iv) whether the causal interaction is mechanically simple (depending on physical contact between cause and effect) or involves hidden forces; and (v) the range of physical variables whose causal effect is understood. There is a range from cases where the causal understanding is tied to a specific context and/or task, to cases in which the understanding is decoupled from a specific task and may be extrapolated to new ones. Furthermore, understanding causal structure facilitates producing an opportunity (phase 6, table 1; e.g. making a tool to obtain out-of-reach food).

## 4. Interactions between components and mechanisms: an embodied view on innovation

Interactions with novel opportunities are influenced by the agent's motivational and cognitive systems, as well as by morphological capabilities and its motor repertoire. The embodied view of cognition emphasizes a feedback process between an agent's sensorimotor capacities, motivational and cognitive systems, and affordances [26–28,51]. For example, an animal might encounter a highly novel resource, yet have no knowledge about its potential. Exploitation of an opportunity of this kind will only be available to an agent that has a spontaneous inclination to explore novelty within its environment (e.g. house sparrows (*Passer domesticus*) discovering how to use a sensor to open automatic doors [33]). Alternatively, a resource might strongly resemble an item in an agent's existing range of resources. This kind of opportunity will be available to a larger range of agents, including those more wary of novelty.

The affordance of the opportunity also plays a role in this interaction [52,53]. Consider the difference between an eruption of rabbits and of porcupines into a carnivore's range. Porcupines offer fewer affordances, and in general, they are targets that can be exploited in only a few, very specific and error-intolerant ways. Low affordance opportunities are more likely to be exploitable by those animals equipped with broad behavioural repertoires, as they are more likely to have at their disposal pre-existing motor action patterns that can be applied to these novel resources. Hard-shelled prey, for instance, have a low affordance because they can be exploited only by breaking the shell. If power is required, then access will be further restricted to those with broad behavioural repertoires and strength. Opportunities that can only be exploited by using a combination of motor actions (e.g. holding, ripping and smashing) will also most likely be available exclusively to those agents with high motor diversity. Alternatively, success might be possible for those capable of applying their motor actions to a novel context (e.g. dropping prey on to a hard surface by an individual that already drops other foods). This level of motor generalization might be underpinned by a perceptual similarity between the new case and familiar ones (dropping/crushing *hard* foods) or through an abstract rule (hard objects break only through forceful contact with another hard surface).

Another combination of opportunity and behavioural repertoire is one in which an agent with a limited behavioural repertoire interacts persistently with an opportunity with many action possibilities (i.e. high affordance). This is more likely if the potential of the resource is already known to the agent and the reward is high. Sugar packet opening by Barbadian bullfinches (*Loxigilla barbadensis*) [54] and milk bottle opening by blue tits (*Cyanistes caeruleus*) [55,56] are examples where a resource that can be accessed in multiple ways was discovered opportunistically (either from individual experience or from watching others) in its readily available form. After the initial discovery in the available form, the opportunity is then accessed through a barrier by individuals persisting in the expectation of reward.

Finally, the environment itself can support innovation. For instance, young chimpanzees (*Pan troglodytes*) might try to crack open nuts on all kinds of surfaces but the inherent properties of a stone anvil offer the fastest route to success for opening nuts; therefore, the use of stones as anvils is reinforced.

## 5. Phases of innovation

We suggest that innovation processes can be decomposed into six phases (table 1) characterized by different combinations of the structural components proposed above (figure 1).

1. *Discovery of an opportunity*. A novel (potential) opportunity is discovered by behavioural interaction or by perception only; this does not necessarily imply that its immediate benefit or use is also discovered.

*Mechanisms*: directing attention to a novel aspect of the environment, neophilia, being attracted by a novel aspect of the environment, play [57] and chance. Note that for this and the subsequent phases the list is of mechanisms not necessarily complete.
*Acquired knowledge*: recognition of potentially relevant stimuli (i.e. the novel opportunity is noticed).

2. *Initial discovery of a favourable behavioural interaction that makes an opportunity accessible*. Through interaction, the agent acquires knowledge about this opportunity, its benefit, and about the relationship between its behavioural pattern and the beneficial effect of the opportunity.

*Mechanisms*: neophila, explorative behaviour, play, motor diversity, attention to the parts of the environment that change with the interaction, persistence, flexibility and chance.
*Acquired knowledge*: stimulus–response contingencies, i.e. preliminary knowledge of the potential beneficial effect of the opportunity.

3. *Repetition and testing*. This phase results in a decrease of uncertainty about the existence of a beneficial relationship between behavioural pattern and opportunity (i.e. a gain in knowledge about this relationship, see above). The knowledge gained as the agent interacts with the opportunity allows minor adjustments leading to an optimization of the behavioural pattern.

*Mechanisms*: cognitive mechanisms including association, optimization, stimulus generalization, causal reasoning, trial-and-error strategies. The association can be formed from direct interaction with the environment or in the mental domain only (e.g. by combining existing knowledge).
*Acquired knowledge*: stimulus–response contingencies, knowledge of statistical covariance, which can gradually result in causal knowledge.

4. *Social diffusion*. The knowledge about this novel interaction with an opportunity (or only about the opportunity) spreads through the population. As is argued by Reader & Laland [2] and Price [58], evolutionarily relevant and significant innovations have to spread in the population and be transmitted inter-generationally. Three paths of diffusion can be observed:

(i) Behavioural innovations can be acquired by social interaction (e.g. by observation) with others and spread this way in the population. Social learning is an important process for the acquisition and diffusion of innovations in humans and animals [59]. However, social transmission depends on the likelihood that agents tolerate each other and interact near novel resources, and thus on social organization. The speed of social transmission also depends on whether information is spread only between generations from parents to offspring or also within one generation.
(ii) Several behavioural innovations may arise independently and then spread from these original sources by social learning. In this case, innovation spreads through a combination of individual and social learning (e.g. milk bottle opening by blue tits [55,56]). However, if a novel opportunity arises and the species has the cognitive and morphological abilities to access it, it is possible for many individuals of a population to converge even without social learning [60].
(iii) Finally, if an innovation is already widespread and has high adaptive value, it might become gradually assimilated into the genome; tool use in the woodpecker finch (*Cactospiza palliida*) is a potential example [58,61]. Genetic assimilation (the ‘Baldwin effect’) will facilitate the progress to all subsequent phases of the innovation process since it provides a higher base level from which future generations can start [62].
  *Mechanisms*: all of the above for repeated independent innovation, all mechanisms of social learning.
  *Acquired knowledge*: knowledge is acquired through observation of other agents or through individual interaction with the opportunity. Agents learn stimulus–response contingencies, knowledge of statistical covariance. This can gradually result in causal knowledge.

5. *Extension: exploitation of an adjacent opportunity*. This phase involves using the behavioural pattern and/or the acquired knowledge to actively exploit another opportunity. The novel opportunity can be found in the same domain (e.g. chimpanzees accustomed to using sticks to extract the heart of palms discovered how to use them to spear bush babies out of tree holes [63]) or in a different domain (e.g. exploring feared objects with a stick previously used to extract prey, as with New Caledonian crows [64]). Alternatively, innovations that facilitate colonization of new habitats and niches encourage encountering further opportunities (e.g. potato washing brings Japanese macaques (*Macaca fuscata*) closer to estuary fringes and therefore into contact with yet further opportunities [62]).

The discovery of adjacent opportunities will be more likely if the acquired knowledge is more general (e.g. knowledge about causal structures), for then the agent's exploration process will not be random. However, the discovery of adjacent opportunities could also be possible with random search and learning, as long as the opportunities are obvious*,* costs of exploration are low, and there are some rewards for small variations of established practices.

*Mechanisms*: all of the above and all forms of learning (e.g. operant conditioning and stimulus generalization) as well as cognitive mechanisms that lead to causal knowledge about visible or invisible mediating forces (causal reasoning).
*Acquired knowledge*: stimulus generalization allows an agent to build a more general category of the opportunity (e.g. not only water but all kinds of liquids can be collected with a leaf) or generalize the consequences of a behavioural interaction (e.g. as with nuts, a tortoise can be cracked by dropping it because both nuts and tortoises are hard (perceptual similarity) or because they are both food (categorical similarity)).

6. *Producing: reproduce/create the discovered opportunity*. The knowledge acquired from the interaction is used to reproduce the opportunity (e.g. shifting from accidental discovery and use of fire to *actively making/producing* fire; or New Caledonian crow tool manufacture [65]). If the initial innovation is established as part of the behavioural repertoire of a population and not just of an individual agent, this process is further facilitated and accelerated, as this initial innovation acts as a basis for its further development. This step could also be facilitated if the original innovation is already genetically entrenched: the production can start from this genetic base [62].

*Mechanisms*: the mechanisms are similar to those of phase 5 plus potentially planning. Phase 6 innovations will also be aided by social transmission of the initial innovation, since an incremental innovation is much more likely once the innovation has spread through the local group.
*Acquired knowledge*: generalized knowledge about the opportunity and knowledge about how to (re-)produce this opportunity.

**Figure 2.**
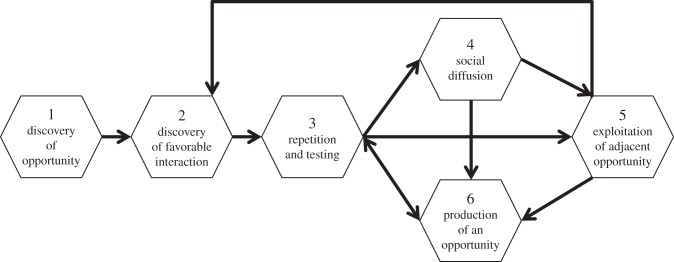
Overview of the dynamics of the phases of innovation. These phases form a cyclical process.

One has to keep in mind that these phases do not necessarily follow the proposed linear order. Rather, some phases might be skipped and, more importantly, the whole framework has to be thought of as a *cyclic system*. For instance, the result of phase 5 may act as a starting point for phase 2 (figure 2). Hence, a ‘trajectory of innovations’ might evolve over time and lead to increasingly complex innovations.

## 6. Consequences for fitness and macroevolution

In the view developed here, innovative behaviour is a special case of niche construction. Niche construction theory [66] has emphasized the active role that agents take in shaping not just their physical environment but also their selective environment, and how this reshaping can affect fundamentally the evolutionary trajectory of a lineage. In effect, our framework for thinking about innovation shows how variations in some of these niche-constructing activities arise, and thus alter these evolutionary trajectories differentially. We propose that the innovations themselves drive evolution (though only when they have significant immediate fitness effects) because they initiate feedback cascades that then expose the underlying mechanisms to new selective forces. If random genetic variation generates a less costly mechanism for achieving the same innovation (allowing a fitness gain), then the latter mechanism will be selected. However, it is not the mechanism *per se* that is under selection, but rather the phenotype via its tally of costs and benefits that result in a given fitness output.

According to our framework, variations in several aspects of the innovation process determine their effects on the selective environment of an agent or a population. The following aspects determine the consequences for fitness and macroevolution.

(i) Importance of the resource. This aspect depends both on quantity and quality. An opportunity that can be exploited frequently has a larger fitness footprint than one that can be exploited occasionally. However, a resource that is less frequently available might have huge consequences for fitness. Examples include discovering an opportunity–behavioural interaction in times of famine, or finding something to drink on an extremely dry island, as in the case of blood drinking by the sharp-beaked ground finch (*Geospzia difficilis*) [67].
(ii) Number of innovations within a population. If a taxon consists of individuals with a high propensity for innovation (e.g. Darwin's finches and corvids [68,69]), colonization of new habitats and the creation of new niches will be facilitated, which in turn promote radiation. For instance, the high rates of feeding innovations by Darwin's finches allowed them to colonize remote and hostile islands in the Galápagos archipelago and led to geographical isolation, which is an important step in the radiation process [70].
(iii) Level of diffusion into the population; that is to say, whether or not an innovation is transmitted socially to other agents in the population. The more widespread an innovation is within a population, the higher is its evolutionary relevance.
(iv) Generalizability of application. The broader the scope for application of the innovation, the greater the potential for the innovation to expand to further fitness-enhancing opportunities.
(v) Multiplication of opportunity. The production of an opportunity reduces a population's dependence on its natural occurrence. This greatly expands the capacity to penetrate new niches, as shown for the production of fire by humans [71].

## 7. Theoretical and practical advances provided by the framework

The utility of distinguishing different levels of innovations is in (i) recognizing that innovations differ in terms of their underlying mechanisms and their evolutionary consequences, (ii) proposing a framework that operationalizes the parameters upon which different phases of innovations can be distinguished, and (iii) guiding research into the mechanisms that are involved in different levels of innovation; this could explain their taxonomic distribution. These mechanisms include, but are not limited to: extended developmental periods and play; neophilia; social tolerance; large baseline behavioural repertoires; and the cognitive mechanisms that elevate innovation rate. Research on function cannot fully inform mechanisms, and vice versa. However, our framework demonstrates that the two levels are linked because different mechanisms have different functional consequences.

### (a) Taxonomic distribution and relationship with taxonomic radiation

First, our framework predicts that innovations will vary in their potential to facilitate access to yet more novel opportunities and even new ecological niches, which will in turn lead to variation in rates of taxonomic radiation. This prediction can be tested by estimating the extent to which innovations are likely to generalize from one opportunity to another and testing whether this likelihood correlates with diversification rates. We suggest that innovations that are about the discovery of novel food sources are likely to be relatively narrow in their application (e.g. eating human vomit by rooks (*Corvus frugilegus*) [33], blood drinking in the sharp-beaked ground finch (*Geospzia difficilis*) [67]). Characteristics that make innovations more widely applicable are those that involve novel motor patterns (because novel actions become available to be used in response to other novel opportunities) and those that incorporate objects into behavioural patterns (because objects become available to be used in response to other novel opportunities). In other words, both novel behavioural patterns and tools (broadly defined) have the potential to shift in their use. Such innovations can be readily identified and counted, and could be related to rates of taxonomic radiation. Studies that have tested the relationship between innovation and lineage diversification have correlated numbers of innovations and species richness [6–10]. According to our model, the number of innovations is a sensible measure because many independent innovations facilitate the colonization of new habitats. Our framework extends previous work by proposing that radiations will depend not only on frequency of innovations, but also on their kind as described above. To test this idea, different types of innovations could be correlated with speciation rate to assess their contribution. Other studies have already successfully applied similar distinctions to assess relationships with brain size and ecological parameters (e.g. [11,13,68]).

The framework also indicates that the probability of reaching different phases of innovation will vary depending on the combination of components (opportunity, interaction, acquired knowledge) and their underlying mechanisms. We propose that reaching the initial phases of innovation (table 1: discovery of the opportunity, discovery of an interaction) will depend to a large extent on mechanisms such as attention, motor diversity and the agent's motivational systems. Reaching further phases will depend on these same mechanisms, but also on the breadth of application of the acquired knowledge (see §3c). Diffusion through the group depends, of course, on social organization and propensities for social learning. Finally, we predict that ‘producing an opportunity’ (phase 6) will be achieved more frequently in animals that possess complex cognitive abilities.

Our framework suggests additional reasons why the frequency and taxonomic distribution of innovation vary [3–5]. An innovation that arises from random motor variation might come at little cost and suffice in many situations to produce novel actions in response to environmental change. Such innovations that depend only on cheap trial-and-error learning will be both taxonomically widespread and will often arise independently within a population. Innovations that depend on multiple mechanisms might come at much greater costs (e.g. large brains, long developmental trajectories, extended parental care) and therefore arise less frequently within one population and with a limited taxonomic distribution. Other sources of variation are the ecological conditions and the levels of environmental heterogeneity to which species and populations are exposed. For instance, it is plausible that a harsh and unpredictable environment, and high environmental heterogeneity, will select for the mechanisms that make innovations more likely.

## 8. How does novel behaviour emerge?

In the morphological literature, novelty is often seen as a problem. This is because it is not clear how a novel morphological feature can arise in small increments from a feature that already exists. How could one go from an unsegmented to a segmented body in incremental, adaptive steps? The challenge is to either show that there is an incremental pathway or to show that a large-step change is not biologically implausible. The same ‘evolution of novelty’ problem applies to the evolution of novel behaviours; some forms of innovations seem to ‘emerge from nowhere’.

For morphology, a lineage explanation has been put forward arguing for evolution in incremental steps. Calcott [72] proposed that lineage explanations have two requirements, namely production and continuity. The production requirement makes explicit the idea that each stage in the lineage must be functional; the continuity requirement rules out large jumps from one step to the next. In contrast to morphological evolution, the continuity requirement is not so important in the case of behavioural innovations because behaviour sometimes produces large variations, not just small and intermediate ones.

The difficulty of explaining the emergence of novel morphologies and novel behaviours overlaps in the production requirement. Although a stepwise development via adaptive stages is recognizable in many examples of animal innovation, especially when an existing behaviour is used in a novel context or novel behaviour patterns result from a combination of existing ones, it is still difficult to explain the origin of complex behavioural patterns. This is particularly so in examples where incomplete sections of the behavioural sequence are not rewarded, e.g. tool use in the woodpecker finch. This species inserts twigs into tree holes to extract prey, a process that requires complex motor coordination. Even though this behaviour is now partly genetically entrenched [69], genetic entrenchment begins with an individual innovation in a rudimentary form. How were these rudimentary forms sufficiently rewarded to stabilize the behavioural platform from which the current behavioural complex emerged? Even if ancestral woodpecker finches were predisposed to manipulate sticks (e.g. for nest building), it is hard to imagine how they first came to poke a stick into a tree hole and even more difficult to imagine how such an accidental, rudimentary behaviour led to reward. Thus, if we model an innovation as the result of incremental, associative learning in an individual agent, the production requirement (i.e. each stage has to be adaptive) is not fulfilled.

The lineage explanation addresses this problem on the phylogenetic level, but the same constraint holds on the ontogenetic level if we consider that a complex skill is acquired through successively reinforced steps. The solution is that for some agents, reward is not always external reward. We propose that motivational systems–particularly exploration and play–and cognitive mechanisms–learning, social referencing and particularly causal reasoning–provide the bridge between intermediate steps, some of which may not be adaptive in themselves. Exploration and, to an even greater extent, play are two motivational systems leading to novel combinations that can be repeated over and over again without the requirement for any extrinsic reward. These mechanisms can produce potentially adaptive behaviour. Although play generally fades away, many adults continue to explore and some, particularly those with larger brains, continue to play, albeit at reduced frequencies [73]. Spontaneous emergence of stick tool use and tool fabrication has been recently observed in Goffin's cockatoos (*Cacatua goffiniana*) [17] and keas (*Nestor notabilis*) [16], both of which show extensive object play even in adulthood. The prediction that innovation is related to play could be tested by relating the taxonomic prevalence of play, separated into juvenile and adult play, to innovation measures.

Understanding to some extent how and why one event causes another allows an agent to appreciate and anticipate the beneficial consequences of an opportunity, a novel interaction pattern or a novel object even without immediate reward. Causal knowledge can bridge ‘non-functional’ unrewarded stages and, as with play and exploration, causal knowledge facilitates the discovery of adjacent possibilities (e.g. applying a foraging tool to bodily care). Changes in function can promote large-step changes.

The agent's cognitive mechanisms and motivational systems also interact with the affordances of the opportunity. Opportunities can have structural features that make successful interaction with an opportunity more likely, as discussed above in the example of anvil use in chimpanzees.

In sum, we use Calcotts' [72] framework of morphological change to propose an account of the emergence of complex behavioural innovations. The motivational systems, the cognitive mechanisms and the affordances of the opportunities are the components that produce behavioural innovations, and these components remain identifiable from one step to the next. In morphological lineage explanations, the analysis identifies components or structures that are internal to the agent. In contrast to morphological evolution, we propose that the mechanisms that produce adaptive behaviour include the affordances available in the agent's environment. These contribute to generating behavioural novelty. Finally, in contrast to morphological evolution, exploration, play and causal reasoning may have the potential to bridge non-functional stages, finessing the requirement that every stage has to be adaptive.

### (a) How can innovative propensities be measured experimentally?

Field observations do not offer the possibility of investigating underlying mechanisms. Experiments are critical. In experimental studies, innovation is operationalized as success in a given task, most often problem-solving [14]. Individual traits of solvers and non-solvers are then compared, examining characteristics such as age, social dominance, neophobia, reversal learning and motor diversity to identify traits that facilitate innovative behaviour. The problem often involves opening a container to access food. However, it remains unclear whether these tasks tap all relevant aspects of innovativeness. For example, in most experimental tasks used to date, innovation is driven by hunger. As a consequence of this design, innovation is goal-directed and the problem and solution are pre-defined by the task. These tests do not sample the organism's propensity to express novel behavioural patterns spontaneously via non goal-directed exploration of the environment (though see [34]). We propose that different experimental assays will be needed depending on whether interest is purely in delimiting underlying mechanisms or in linking innovation to its functional outcomes. In the latter case, research methods should focus on quantifying aspects of the innovations that are relevant to fitness or macroevolution (e.g. its capacity to invade a new niche). Our framework could help to determine which phase of innovation is most relevant to the question of interest as well as which components and mechanisms are mostly involved in that phase. For example, one might be interested in comparing clades that have radiated and clades that have not radiated with respect to those mechanisms that facilitate finding new opportunities (phase 5, table 1).

An additional research direction would involve manipulating components and underlying mechanisms systematically to assess their importance in the innovation process. The effects of these manipulations should then be tested by within and between species comparisons.

#### (i) Manipulating the opportunity

According to our framework, if the agent knows the potential value of the opportunity prior to innovating, innovation may depend only on motor flexibility and goal-directed motivation. But if not, innovation depends also non-goal-directed exploration and play. One straightforward way to control whether the potential of an opportunity is known in advance involves manipulating visual access to the reward. Discovery of a hidden reward can be used to operationalize innovation without the presence of a reward operating as a driver for exploration, object interaction and changes in motor diversity. Hence, with a small change in experimental methodology (using hidden rather than visible rewards), one can measure the propensity of an animal to encounter (or not) new opportunities through non-goal-directed exploration, interaction and motor variability. Another important parameter identified by our framework, task affordance, could be manipulated by increasing the number of different ways in which a task can be solved. This could be achieved by designing an array of pre-defined manipulation options to reach a given goal, as in the multi-access box 16, or alternatively, by providing a task in which the agent is free to express as much motor variation as it chooses to or is capable of [21,74].

A parallel line of research would involve beginning to measure generalization of acquired knowledge. Based on the arguments developed here, this could be achieved by providing agents with the opportunity to innovate and then measuring their performance on a variety of perceptually similar/different/very different tasks, while controlling experimentally or statistically for potential changes in motivation that might differ between solvers and non-solvers. Different tasks could require the same motor action in perceptually very different tasks (e.g. lifting is key to solving).

#### (ii) Manipulating knowledge acquisition

Rather than testing generalization, a parallel line of research could involve training agents to generalize from one innovation opportunity to another and sampling inter-individual and inter-species propensity in this trait. This could be achieved by training them on a series of transfer tasks. Our assumption is that training on one task makes knowledge more specific but training on multiple tasks could give rise to a broader knowledge base from which to generalize. For example, one could train an agent to drop various objects into a tube to obtain a reward and test whether the propensity to generalize to other new objects increases.

In addition to manipulating the components and mechanisms of innovation, we suggest that it will be important to test the phases through which the innovation subsequently proceeds. Hence, generalization and social diffusion studies should become an integral part of studies of innovation as these affect the fitness and macro-ecological consequences of novel behaviour.

## 9. Conclusion

Innovations are currently considered a broad behavioural category explained proximately by cognitive mechanisms, and explained functionally by their adaptive payoffs in harsh and changing environments. We have attempted to describe the complexity of innovation mechanisms and to build a framework that illustrates the interaction between mechanism, fitness benefit and evolutionary significance, using the information gained through interaction as an intervening variable. In doing so, our framework splits innovation into several separate units (components and phases) that can be investigated experimentally. Decomposing innovations in this way also reveals the complex interaction between environment, behaviour and underlying mechanisms. Innovations are diverse not just in arising from different mechanisms, but in their consequences for fitness and macroevolution. Finally, our framework articulates how this diversity can be studied systematically. We suggest that the most interesting research directions will involve exploring the interactions identified by our framework, namely those between the diversity of mechanisms, the breadth of the knowledge gained and the adaptive significance of innovation.
